# Excellent clinical and radiological outcomes after arthroscopic reduction and double row‐suture bridge for large‐sized greater tuberosity fractures of the humerus

**DOI:** 10.1002/ksa.12506

**Published:** 2024-10-15

**Authors:** Sang‐Hun Ko, Jaemin Oh, Ki‐Bong Park, Sangheon Oh, Young Dae Jeon

**Affiliations:** ^1^ Department of Orthopaedic Surgery, University of Ulsan College of Medicine Ulsan University Hospital Ulsan Republic of Korea

**Keywords:** arthroscopic reduction, double‐row suture bridge, greater tuberosity fracture, large‐sized

## Abstract

**Purpose:**

Currently, there is limited information on the clinical outcomes of arthroscopic reduction and double‐row suture bridge fixation for large greater tuberosity fractures of the proximal humerus. This study aimed to evaluate the radiological and clinical outcomes of arthroscopic reduction and double‐row suture bridge fixation for these fractures, hypothesizing that arthroscopic reduction and double‐row suture bridge fixation is a safe, effective and minimally invasive treatment for large greater tuberosity fractures.

**Methods:**

This retrospective study analysed patients with large greater tuberosity fractures (fracture fragment ≥30 mm in diameter) who underwent arthroscopic reduction and double‐row suture bridge fixation and had a follow‐up period exceeding 2 years. The anatomic reduction was confirmed by assessing the step‐off on radiographs immediately after surgery, and the radiologic union time was recorded. At the final follow‐up, range of motion and functional outcome scores were evaluated. Additionally, any surgery‐related complications were evaluated.

**Results:**

Fifteen patients with a mean follow‐up of 57.7 ± 23.1 months were included in the study. The mean fracture fragment size was 32.5 ± 2.4 mm, with a mean displacement of 5.1 ± 1.6 mm. Immediately postsurgery, 13 of 15 patients (86.7%) had a fracture step‐off of <3 mm, with an average union time of 3 months. At the final follow‐up, patients demonstrated excellent outcomes, with an average forward flexion of 167 ± 9.7° and external rotation of 70 ± 16.3. Functional outcome scores showed significant improvement compared with preoperative scores (*p* < 0.001). No major surgery‐related complications were reported.

**Conclusions:**

Arthroscopic reduction and double‐row suture bridge fixation for large‐sized greater tuberosity fractures is safe and shows good fracture reduction and excellent clinical outcomes. Therefore, this surgical method can be considered an alternative to open reduction for large greater tuberosity fractures.

**Level of Evidence:**

Level IV.

**Abbreviations**
ASES: American Shoulder and Elbow Surgeons
ULCA: University of California‐Los Angeles
VAS: Visual Analog Scale

## INTRODUCTION

Greater tuberosity fractures of the proximal humerus represent approximately 20% of all proximal humeral fractures, with only 5% requiring surgical intervention [4]. The presence of greater tuberosity fracture and dislocation of the anterior shoulder joint has been reported in 5%–57% of cases [1, 4, 10]. Traditionally, surgical intervention involved open reduction and internal fixation, but recent studies have shown promising results with an arthroscopic approach [2, 7, 8, 13, 15, 24].

Arthroscopic surgery offers several advantages, including easy visualization, the ability to mobilize fracture fragments, and a suitable field of view for addressing intraarticular lesions or bipolar lesions of the greater tuberosity and glenoid fossa [17]. This makes it particularly beneficial for fixing small fracture fragments [29]. Additionally, many greater tuberosity fractures are accompanied by partial‐thickness rotator cuff tears, and arthroscopic surgery allows for concurrent rotator cuff repair, adding to its versatility [11, 19, 21, 30]. However, arthroscopic fixation also has its challenges. It requires a long learning curve, and the surgeon's experience and skill level significantly impact the surgical outcome. Moreover, the operation time is generally longer than that for open surgery [18].

Despite these challenges, previous studies have reported good results with arthroscopic reduction and fixation for small‐sized greater tuberosity fractures [7, 15]. Although surgical techniques for arthroscopically managing large‐sized fractures have been described [9], the clinical outcomes for large greater tuberosity fractures with fragments >30 mm have not yet been reported.

Therefore, this study aimed to assess the radiological and clinical outcomes of arthroscopic reduction and double‐row suture bridge fixation for large greater tuberosity fractures of the proximal humerus. By evaluating these outcomes, this study hypothesized that arthroscopic reduction and double‐row suture bridge fixation would prove to be a safe, effective and minimally invasive treatment option for managing large greater tuberosity fractures.

## MATERIALS AND METHODS

This retrospective study was approved by our Institutional Review Board (IRB No. 2022‐06‐009‐001). The requirement for obtaining informed consent was waived. A retrospective analysis was conducted on 52 patients who underwent arthroscopic reduction and double‐row suture bridge fixation for greater tuberosity fractures between February 2014 and February 2021. Arthroscopic reduction and double‐row suture bridge fixation were performed in cases where displacement exceeded 5 mm or 3 mm for patients engaged in hard labour.

### Inclusion and exclusion criteria

Among the 52 patients who underwent arthroscopic reduction and double‐row suture bridge fixation for avulsion and split‐type humeral tuberosity, those with bone fragments >30 mm on three‐dimensional computed tomography and with follow‐up for over 2 years were included. Exclusion criteria were bone fragment size <30 mm, ipsilateral glenohumeral joint arthritis, rheumatoid arthritis, posttraumatic arthritis, history of previous surgery and those receiving worker compensation.

The displacement and size of the fracture fragments were measured using three‐dimensional computed tomography prior to surgery. Of the total patients, 30 had large greater tuberosity fractures with a diameter >30 mm. Among these, 15 patients with a postoperative follow‐up period of >24 months were selected for the study.

### Surgical procedure

**Figure 1:**
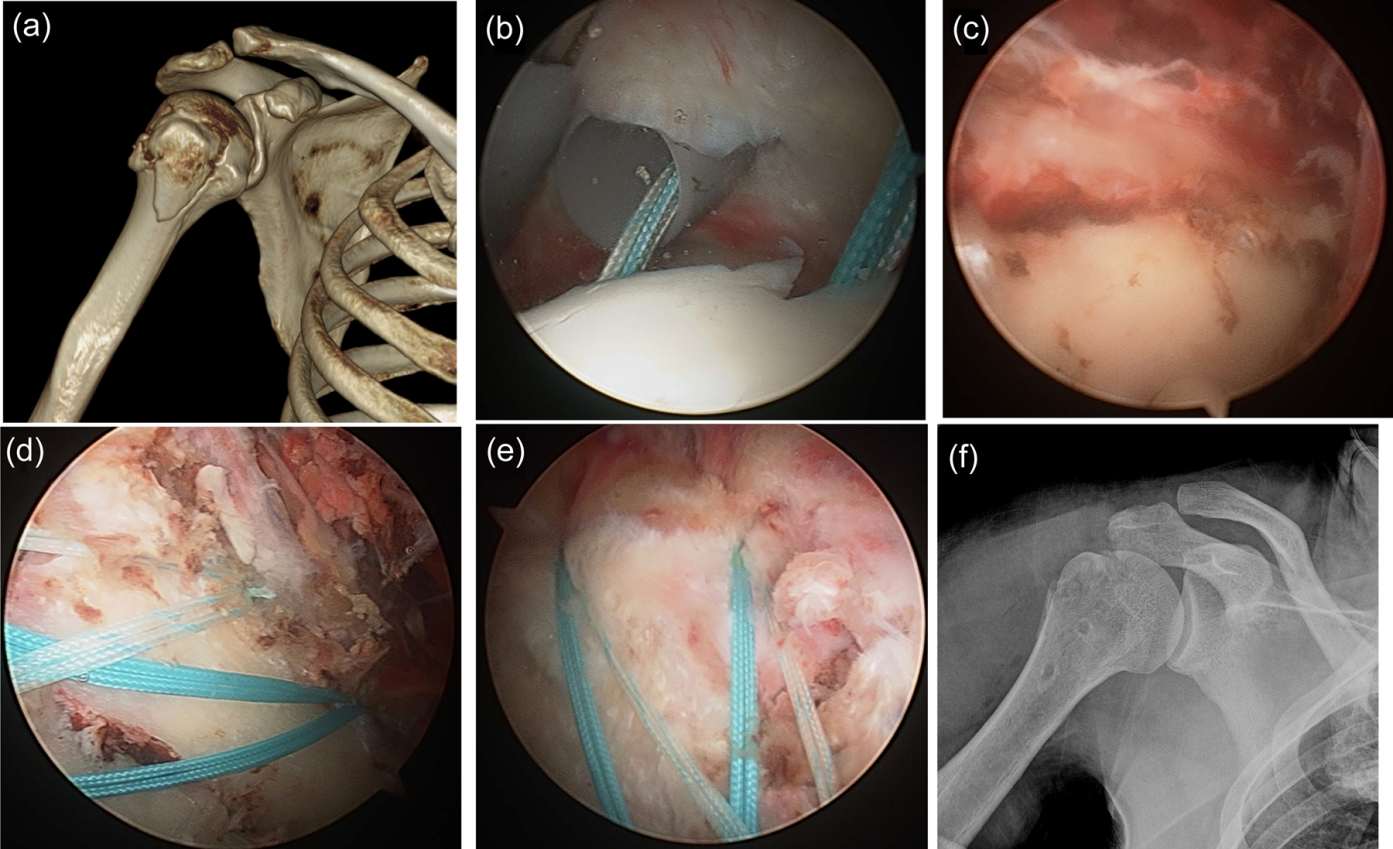
Arthroscopic reduction and double‐row suture bridge fixation for large greater tuberosity fractures of the proximal humerus. (a) Three‐dimensional computed tomography confirming a large greater tuberosity fracture. (b) Insertion of two double‐loaded suture anchors into the medial side of the fracture fragment through the trans‐cuff portal. (c) Verification of the fracture site in the subacromial space. (d) Achieving double‐row suture bridge fixation by placing two lateral anchors into just posterior to the bicipital groove. (e) Demonstration of knotless fixation on the medial side of the fracture site. (f) Immediate postoperative radiograph showing a well‐reduced fracture site.

**Figure 2:**
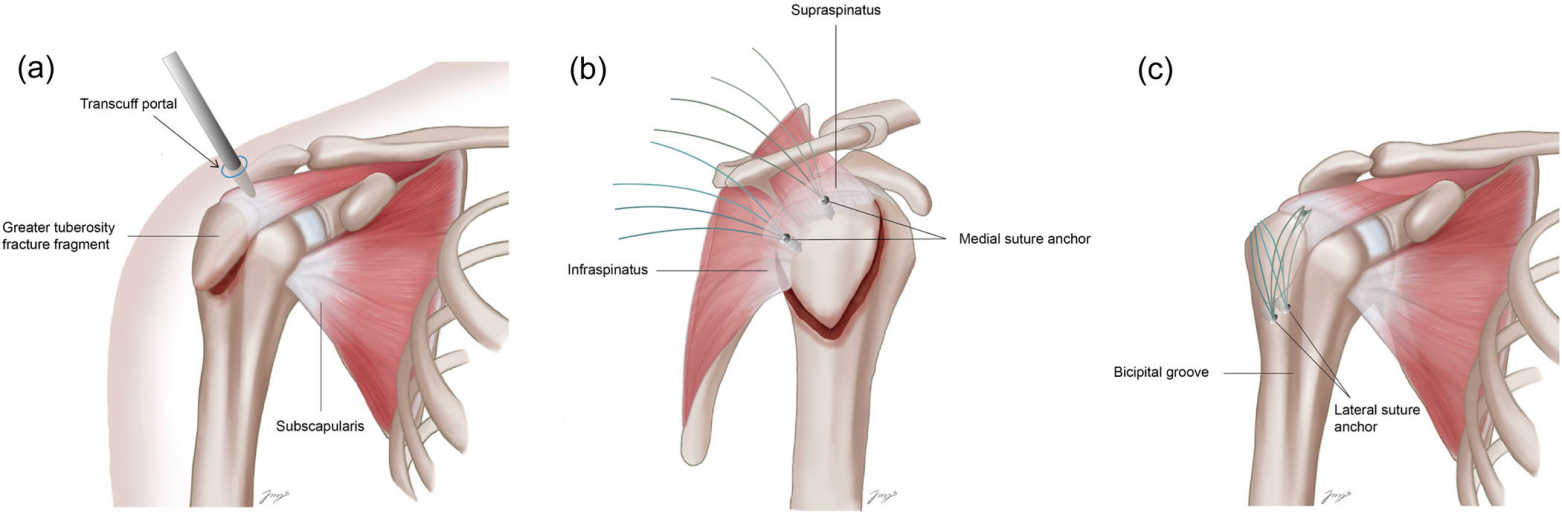
Illustration of arthroscopic reduction and double‐row suture bridge fixation. (a) Insertion of two double‐loaded suture anchors into the medial side of the fracture fragment through the trans‐cuff portal. (b) Eight suture strands from the two double‐loaded suture anchors passing through the intact rotator cuff tendon. (c) Placement of two lateral anchors just posterior to the bicipital groove.

All surgical procedures were performed by two surgeons (S. H. K. and Y. D. J.), with the patients in a beach chair position under general anaesthesia. The joint was inspected using an arthroscope inserted through a standard posterior viewing portal. A trans‐cuff portal was then created, and two double‐loaded suture anchors were inserted on the medial side of the fracture site. For fractures involving the entire greater tuberosity, an anchor was placed in the articular cartilage area nearest to the fracture. All suture strands were passed through the intact rotator cuff tendon using the shuttle relay technique. The arthroscope was then moved to the subacromial space, where a bursectomy was performed to improve visibility. The fracture site was identified, and the haematoma was removed using a motorized shaver. With the strands passing through the rotator cuff confirmed, fracture site reduction was achieved using a Freer elevator and switching stick. Medial knot tying was avoided, and the knotless technique was employed in all cases. Next, the location for the lateral anchor was selected to ensure maintained reduction. Two lateral anchors were inserted just posterior to the bicipital groove of the proximal humerus using a double‐row suture bridge technique, providing a buttress for the fracture fragment (Figures 1 and 2). Finally, arthroscopy confirmed that the reduction was maintained during internal and external rotation of the humerus.

### Outcome assessment

All outcome assessments were performed by two fellowship‐trained shoulder surgeons. Radiological outcomes were assessed by evaluating fragment step‐off on immediate postoperative radiographs to confirm anatomic reduction. Intraobserver reliability and interobserver reliability were assessed. The timing of bone union was confirmed, with radiological union defined as complete cortical bridging between the humeral head and greater tuberosity observed on radiographs. For functional outcomes, the range of motion, Visual Analog Scale (VAS) score, American Shoulder and Elbow Surgeons (ASES) score and University of California‐Los Angeles (UCLA) score were evaluated at the last follow‐up. Active shoulder range of motion was measured using a goniometer for forward flexion and external rotation at the side, while internal rotation was identified based on the vertebral level corresponding to the patient's thumb placement (1–12 for thoracic vertebrae, 13–17 for lumbar vertebrae and 18 for the sacrum). Postoperative complications were also assessed.

### Statistical analysis

All statistical analyses were performed using SPSS v.25.0 software (IBM Corp.). The paired *t* test or Wilcoxon signed‐rank test was used to compare pre‐ and postoperative clinical scores, depending on the results of the Kolmogorov–Smirnov normality test. Statistical significance was set at *p* < 0.05. The intraclass correlation coefficient was used to assess intraobserver reliability and interobserver reliability. The Intraclass correlation coefficient is typically classified as follows: scores above 0.8 denote excellent agreement, scores between 0.6 and 0.8 indicate substantial agreement, scores between 0.4 and 0.6 reflect moderate agreement, scores between 0.2 and 0.4 suggest fair agreement and scores between 0.1 and 0.2 represent slight agreement [20]. A power analysis was conducted using ASES scores to evaluate the adequacy of the sample size of 15 patients. A paired design will be used to test whether the paired difference in distributions (δ) is different from 0 (H0: *δ* = 0 versus H1: *δ* ≠ 0). The comparison will be made using a two‐sided, paired‐difference Wilcoxon Signed‐Rank test, with a Type I error rate (α) of 0.05. The underlying standard deviation of the paired difference distribution is assumed to be 6.8. To detect a paired mean difference of 49 with a sample size of 15 pairs, the power is 1. The power was computed using PASS 2023, version 23.0.3.

## RESULTS

The study included 15 patients (eight men and seven women) with a mean age of 53.9 ± 11.5 years. The average follow‐up period was 57.7 ± 23.1 months. The mean fracture fragment size was 32.5 ± 2.4 mm, and the mean displacement was 5.1 ± 1.6 mm.

Concomitant injuries included shoulder joint dislocation in six of 15 patients (40%) and Bankart lesions in four of 15 patients (27%, with no bony Bankart lesions observed). Supraspinatus partial‐thickness tear rate was nine of 15 patients (60%), with one patient also involving an infraspinatus partial‐thickness tear. The subscapularis tear rate was one of 15 patients (6.7%). No full‐thickness rotator cuff tears were observed. Brachial plexus neuropathy occurred in one of 15 patients (6.7%), with the affected patient recovering 6 months postinjury.

Radiological outcomes revealed seven patients of avulsion‐type greater tuberosity fractures and eight patients of split‐type fractures, with seven of the 15 patients having comminuted fractures. A step‐off of <3 mm immediately after arthroscopic reduction and double‐row suture bridge fixation were observed in 13 of 15 patients (86.7%), and the mean union time was 3 months postoperatively. All measurements were excellent intraobserver and interobserver reliability (Table 1).

**Table 1:** Intraclass correlation coefficients of intraobserver reliability and interobserver reliability.

Variables	Intraobserver reliability*		Interobserver reliability*
	Surgeon A	Surgeon B	
Fragment size	0.989	0.993	0.995
Displacement	0.921	0.932	0.952
Step‐off	0.998	0.995	0.993

* *p* < 0.001.

All final postoperative range of motion and functional outcome scores showed significant improvement compared to preoperative scores, with particularly notable increases in ASES and UCLA scores (*p* < 0.001) (Table 2).

**Table 2:** Statistical analysis of pre‐ and postoperative range of motion and functional outcome scores.

Timing	Forward flexion	External rotation	Internal rotation	VAS	ASES	UCLA
Preoperative	69 ± 15.3	12 ± 12.4	17 ± 1.7	7.1 ± 2.1	43.8 ± 16.4	10.7 ± 3.5
Postoperative	167 ± 9.7	70 ± 16.3	10 ± 2.7	0.6 ± 1.2	92.8 ± 9.6	30.9 ± 4.4
*p* value	< 0.001	< 0.001	< 0.001	< 0.001	< 0.001	< 0.001

*Note*: Internal rotation at the back was identified based on the vertebral level corresponding to the patient's thumb placement (1–12 for thoracic vertebrae, 13–17 for lumbar vertebrae and 18 for the sacrum).

Abbreviations: ASES, American Shoulder and Elbow Surgeons Score; UCLA, University of California‐Los Angeles score; VAS, Visual Analog Scale.

No complications were reported, including cases of nonunion, malunion, or complications related to suture anchors.

## DISCUSSION

Excellent radiological and clinical outcomes were observed with arthroscopic reduction and double‐row suture bridge fixation, even for large greater tuberosity fractures, according to the main findings of our study. Notably, 86.7% of patients achieved a step‐off of <3 mm immediately after surgery, and all showed significant improvements in postoperative functional outcomes. Importantly, no complications were reported.

The surgical indications for greater tuberosity fractures are still a topic of debate. Generally, surgery is considered for displacements of >5 or >3 mm in professional athletes and workers. Conversely, conservative treatment is recommended for depression‐type fractures [4, 25]. The necessity of surgical intervention for displacements between 3 and 5 mm remains controversial, as even a 3‐mm displacement can alter rotator cuff biomechanics and impact functional outcomes [25, 26]. In this study, surgical treatment was performed for displacements of 3 mm or more, particularly in patients engaged in hard labour.

The rotator cuff attaches to the greater tuberosity of the humerus, and in high‐energy trauma, a large greater tuberosity fracture can occur due to the strong contraction of the rotator cuff [4]. The deforming forces from the pull of the rotator cuff muscles should be considered when reducing fracture fragments [3]. Most greater tuberosity fractures involve the supraspinatus and infraspinatus facets, leading to displacement in the posterosuperior direction [26, 27]. Therefore, reduction can be achieved by passing sutures through the intact rotator cuff area.

Previous studies using arthroscopy primarily focused on relatively small greater tuberosity fractures (< 20 mm) [8]. For larger bone fragments, arthroscopic‐assisted plate fixation is sometimes employed [23]. However, our study uniquely addresses larger fractures, with a minimum fracture size of 30 mm and an average size of 32.7 mm. This study is the first to report on arthroscopic surgical outcomes for greater tuberosity fractures with a diameter of ≥30 mm.

In this study, 86.7% of patients had a step‐off of the greater tuberosity fragments within 3 mm immediately after surgery, as confirmed radiographically. While complete anatomical reduction might not always be achieved with arthroscopic surgery compared with open surgery, bone union was successfully achieved in all patients without any radiological complications. Ji et al. reported postoperative residual superior and/or posterior displacement of 0.1 and 0.2 mm, respectively, after arthroscopic surgery in patients with greater tuberosity fractures [7]. However, their study excluded patients with large greater tuberosity fractures. Our study specifically focused on these larger fractures and demonstrated successful anatomical reduction. Previous studies reported postoperative union durations ranging from 8 to 20 weeks [7, 14]. In our study, bone union was achieved in all patients within an average follow‐up period of 3 months. This result is faster than the average union time reported for open reduction surgery [14] and is comparable to the results of a previous study using arthroscopic reduction surgery [7]. Compared with open surgery, arthroscopic surgery minimizes disruption to the soft‐tissue envelope, which accelerates the bone healing cascade and results in rapid bone union. Additionally, it reduces postoperative fibrosis and facilitates rehabilitation, enabling rapid functional recovery [5, 22].

Several studies have demonstrated good functional outcomes with arthroscopic surgical treatment of greater tuberosity fractures. Ji et al. reported successful results by combining metal internal fixation with arthroscopic suturing [23]. However, only two studies have directly compared the outcomes of open reduction surgery and arthroscopic surgery for humeral greater tuberosity fractures [12, 16]. The study found that although arthroscopy had a longer surgical time (95.3 vs. 61.5 min), it resulted in a better range of motion and higher ASES scores. However, in this study, patients with isolated displaced greater tuberosity fractures underwent open reduction when the displacement was >1 cm or the fragment size exceeded 3 cm, whereas arthroscopic surgery was performed for displacements <1 cm or fragment size <3 cm. This discrepancy in surgical indications indicates that the study by Liao et al. cannot be considered an independent cohort study. In their study, patients with fragments 3 cm or larger treated with open reduction had a forward flexion of 137° and an ASES score of 87.4 after a minimum of 2 years. In contrast, our study exclusively performed arthroscopic surgery on greater tuberosity fractures >3 cm. At the final follow‐up, we observed superior outcomes, with forward flexion of 166° and an ASES score of 92.8 compared with the results reported by Liao et al. [16]. Similarly, Yoon et al. achieved an ASES score of 92.6 at 2 years postoperatively using minimally invasive open reduction and internal fixation using a screw and washer for a large greater tuberosity fracture (33 ± 6 mm) [31]. These findings suggest that arthroscopic fixation could be a viable alternative for treating large‐sized greater tuberosity fractures.

A systematic review reported a complication rate of approximately 15% following greater tuberosity fracture surgery [6]. Potential complications included stiffness, continued pain, heterotopic ossification, anchor protrusion/pullout, unplanned implant removal, loss of reduction and malreduction [28]. In contrast, our study found no cases of surgery‐related complications, indicating that arthroscopic surgery may have a high safety potential. However, the small sample size in our study limits its clinical significance, and further large cohort studies are necessary to confirm these findings.

This study had some limitations. First, this was a retrospective study with a small sample size, with only 15 patients followed for more than 2 years. Second, this case series did not allow for comparisons with open reduction interventions. Third, the absence of complications and the favourable outcomes reported may be attributable to the small sample size. Future research should include randomized controlled studies to compare the outcomes of arthroscopic surgery and open reduction based on fracture size.

## CONCLUSIONS

Arthroscopic reduction and double‐row suture bridge fixation for large‐sized greater tuberosity fractures is safe and shows good fracture reduction and excellent clinical outcomes. Therefore, this surgical method can be considered an alternative to open reduction for large greater tuberosity fractures.

## AUTHOR CONTRIBUTIONS

**Sang‐Hun Ko** and **Young Dae Jeon**: Conceptualization. **Young Dae Jeon**: Methodology. **Ki‐Bong Park**: Validation. **Sangheon Oh**: Formal analysis. **Jaemin Oh**: Investigation. **Jaemin Oh**: Data curation. **Jaemin Oh** and **Young Dae Jeon:** Writing—original draft preparation. **Young Dae Jeon**: Writing—review & editing.

## CONFLICT OF INTEREST STATEMENT

The authors declare no conflict of interest.

## ETHICS STATEMENT

This study was approved by the Institutional Review Board of Ulsan University Hospital (IRB No. 2022‐06‐009‐001). The requirement for informed consent was waived due to the retrospective study design.

## Data Availability

Data that support the findings of this study are available from the corresponding author upon reasonable request.
